# Diabetes mellitus is a potential risk factor for aseptic loosening around hip and knee arthroplasty

**DOI:** 10.1186/s12891-023-06376-z

**Published:** 2023-04-05

**Authors:** Yi Deng, Paul N Smith, Rachel W Li

**Affiliations:** 1 Department of Orthopaedic Surgery, Canberra Hospital, Canberra, Australia; 2 Trauma and Orthopaedic Research Unit, Australian National University School of Medicine and Psychology, Canberra, ACT 2601 Australia; 3 John Curtin School of Medical Research, Australian National University, Acton, ACT Australia

**Keywords:** Aseptic loosening, Diabetes mellitus, Arthroplasty, Osteolysis, Inflammation

## Abstract

**Background:**

Aseptic loosening is a leading cause of revision following total hip and knee arthroplasty which is caused by chronic inflammation around the prosthesis. Diabetes mellitus causes systemic inflammatory changes which could increase the risk of aseptic loosening. This study investigated the association between diabetes mellitus and aseptic loosening around hip and knee arthroplasty.

**Methods:**

A case-control study was conducted at a single arthroplasty centre over the seven-year period of January 2015 to December 2021. Cases were defined as any adult patient undergoing revision hip or knee arthroplasty for aseptic loosening. Controls were randomly selected patients undergoing primary total hip or knee arthroplasty during the same period at a 1:4 ratio. Risk factors were compared between the two groups.

**Results:**

A total of 440 patients were included in our study – 88 in the aseptic loosening group and 352 patients in the control group. The odds of having diabetes mellitus in the aseptic loosening group was 2.78 (95%CI 1.31–5.92, *P* = 0.01). Other risk factors were not significantly different between the two groups.

**Conclusions:**

The incidence of diabetes mellitus is significantly greater in patients undergoing revision arthroplasty for aseptic loosening. Further research is required to explore whether this association is indeed causative.

## Introduction

Aseptic loosening remains one of the most common causes of revision for total hip and knee arthroplasty. Arthroplasty surgery is proposed as the final solution of degenerative pathology in the hip and knee joint [1]. As our population ages, so will the number of joint replacements and the number of revision procedures for aseptic loosening. There are several patient-related risk factors which have been demonstrated to increase the risk of aseptic loosening. These include younger patients, high body mass index (BMI) and diabetes mellitus [2–4]. Identifying and treating modifiable patient-related risk factors could reduce the incidence of aseptic loosening and reduce the need for revision arthroplasty, which is associated with significant risk [5].

The pathophysiology of aseptic loosening is underpinned by debris particles being generated at the various implant surfaces. These elicit an inflammatory response, activating osteoclasts and inhibiting osteoblasts, creating an imbalance in bone homeostasis and osteolysis. This bone loss ultimately results in aseptic loosening of the prosthesis, usually necessitating a revision procedure [6]. Numerous inflammatory cytokines have been implemented in the development of osteolysis including PGE2, TNF-α, RANKL, IL-1 and IL-6 [7–9]. These are secreted by macrophages into the local environment and lead to recruitment of a plethora of other immune cells such as fibroblasts, neutrophils and osteoclasts [6].

Diabetes is a complex metabolic disorder which has been demonstrated to alter the immune system in several ways. Emerging evidence has shown that diabetes is also an inflammatory condition that has systemic consequences [10, 11]. It is associated with various cytokines including CRP, TNF-a and adiponectin [12]. IL-6 has also been associated with the development of diabetes [13]. The negative effects of diabetes mellitus on bone health are well documented. Patients with diabetes mellitus have lower bone mineral density due to the increased activity of osteoclasts and inhibition of osteoblasts [14]. In patients with type-1 diabetes mellitus, low levels of insulin and insulin-like growth factor 1 lead to suppression of osteoblast differentiation and activity [15]. In patients with type-2 diabetes mellitus, hyperglycaemia, advanced glycation end products and chronic inflammation lead to altered bony architecture and weakened biomechanical properties [16].

There certainly can be an interaction between the systemic inflammatory effects of diabetes and periprosthetic inflammation leading to aseptic loosening. There is limited evidence in the literature linking diabetes mellitus and aseptic loosening. A study published in 2003 in total knee arthroplasty patients identified a significantly greater risk of aseptic loosening in diabetics compared to non-diabetics [17]. A more contemporary study of 157 revision procedures for aseptic loosening found an association with hyperglycaemia the day prior to arthroplasty but did not find any significant associations with a diagnosis of diabetes. However, a significant limitation is that blood glucose values were only available for 7% of patients, thereby having potential for selection bias [3].

We aimed to describe the prevalence of diabetes mellitus in a contemporary cohort of patients undergoing revision arthroplasty for aseptic loosening and to compare the odds of having diabetes mellitus between patients with aseptic loosening and patients undergoing primary total hip and knee arthroplasty (controls). Our hypothesis is that patients with aseptic loosening have a greater odds having diabetes compared to controls. Our secondary aims are to identify other patient-related risk factors which could contribute to the development of aseptic loosening.

## Materials and methods

A retrospective case-control study was conducted. All adult patients undergoing revision hip or knee arthroplasty for aseptic loosening at a single centre were included as our cases over the seven-year period of January 2015 to December 2021. The diagnosis of aseptic loosening was made using a combination of clinical assessment, radiological investigations and biochemical markers by the consultant orthopaedic surgeon, with fellowship training in hip and knee arthroplasty. The diagnostic criteria was pain with weightbearing around the affected joint with evidence of radiolucent lines and/or areas of osteolysis around the implant on plain x-rays and computerised tomography (CT) scans. Biochemical markers including white blood cell count, C-reactive protein and erythrocyte sedimentation rates all were within the normal range to be eligible for inclusion in the aseptic loosening group. Exclusion criteria were revision patients who did not have evidence of aseptic loosening, prior history of prosthetic joint infection, incomplete medical records or missing medical imaging. Patients undergoing unilateral primary hip and knee arthroplasty for osteoarthritis were included as our controls. Again, the diagnosis was made by the consultant orthopaedic surgeon using clinical and radiological findings. Controls were randomly selected at a 1:4 case to control ratio using a random number generator. Exclusion criteria were bilateral arthroplasty, hip resurfacing, unicompartmental knee arthroplasty or patellofemoral joint arthroplasty or incomplete medical records. Basic demographic data was extracted from the hospital medical records including age, sex, type of procedure, side of procedure, surgeon, past medical history and blood test results. Deidentified patient data was recorded and stored using a standardised spreadsheet. Prospective ethical approval was granted by the ACT Health Research Ethics and Governance Office, Human Research Ethics Committee (ETH.9.07.865).

Demographics, past medical history and pathology results were extracted from patient records during their attendance at preadmission clinic, 1–4 weeks preoperatively. Diagnoses were broadly divided into categories including diabetes, obesity, cardiovascular, respiratory, renal and autoimmune diseases. Diabetes included type 1 and type 2 diabetes mellitus. Obesity was defined as a BMI > 30 kg/m^2^. Cardiovascular diseases included hypertension, myocardial infarction and congestive cardiac failure. Respiratory diseases included asthma, obstructive airways disease and obstructive sleep apnoea. Renal disease was primarily chronic kidney disease. Cancer included any solid organ malignancy, whether metastatic or not. Autoimmune diseases included any systemic autoimmune diseases.

Data analysis was performed using SPSS Version 26 (IBM, USA). Student t-tests were used to compare continuous data and Chi-squared and Fisher’s exact tests were performed for categorical data to identify differences between the two groups. Odds ratios were calculated to compare the two groups and multivariate logistic regressions were performed to control for potential confounders. A *P*-value of < 0.05 was deemed statistically significant.

## Results

### Demographics

**Fig. 1:**
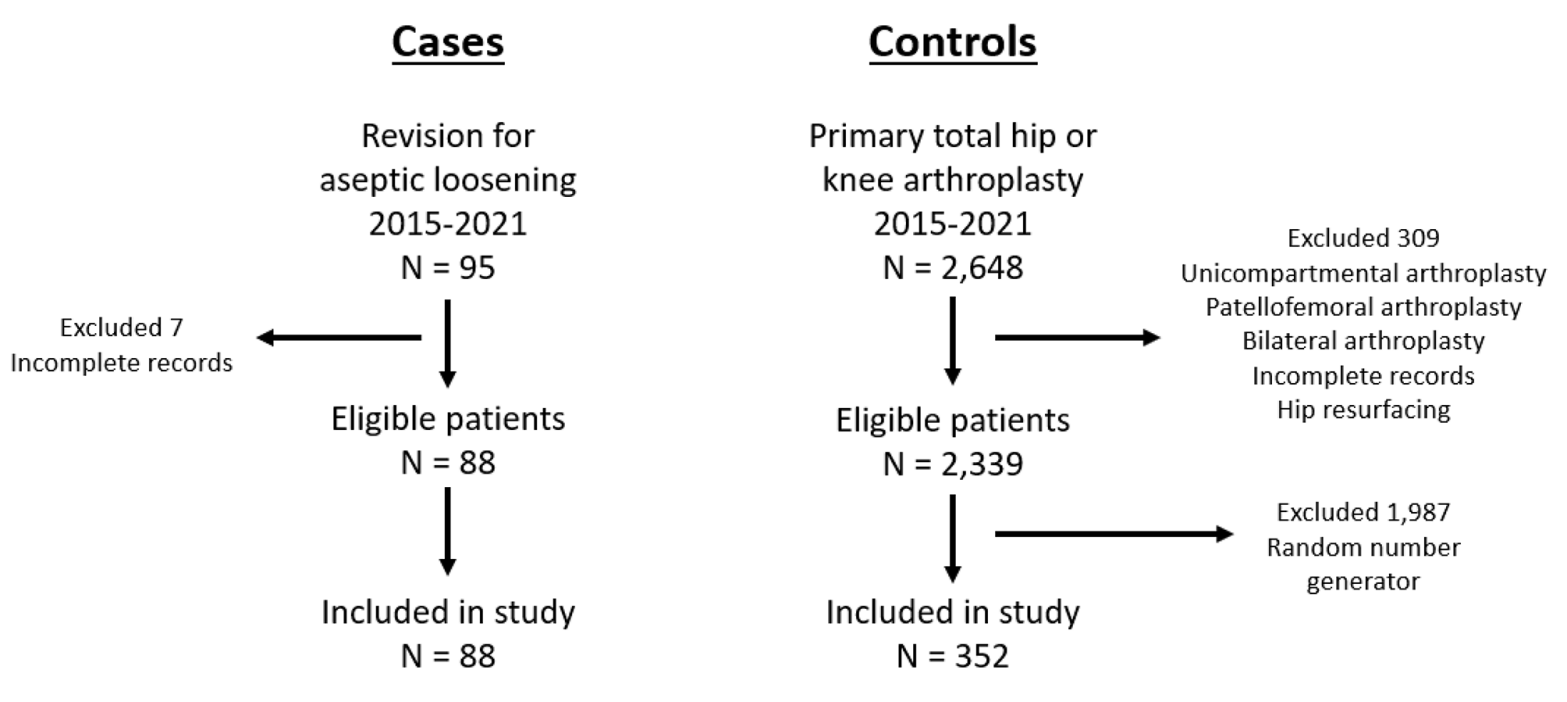
Flow of patients through study

A total of 88 patients undergoing revision arthroplasty for aseptic loosening and 352 controls were included in our study. The mean age was 66 years for the aseptic loosening group and 64 years for controls. There were 57 hips (64.8%) in the aseptic loosening group and 165 hips (46.9%) in the control group. There were more hips in the aseptic loosening group, but otherwise the groups were comparable (Table 1). The flow of patients and exclusion is demonstrated in Fig. 1.

**Table 1:** Patient demographics

	Aseptic loosening (N = 88)	Control (N = 352)	*P*-value
Age (mean ± SD)	66 ± 12	64 ± 11	0.09
Sex (males:females)	35:53	157:195	0.24
BMI (mean ± SD)	28 ± 6	27 ± 4	0.12
Joint (hip:knee)	57:31	165:187	< 0.01
Bloods (mean ± SD)			
Haemoglobin (g/dL)	129 ± 13	133 ± 15	0.56
Calcium (mmol/L)	2.26 ± 0.12	2.26 ± 0.15	0.92
Phosphate (mmol/L)	1.36 ± 0.47	1.10 ± 0.23	0.40
Magnesium (mmol/L)	0.90 ± 0.16	0.87 ± 0.20	0.78
Potassium (mmol/L)	4.60 ± 0.59	4.34 ± 0.23	0.24

### Disease associations

The odds of diabetes were significantly greater in the aseptic loosening group compared to controls (Table 2). Fourteen of 88 patients (16%) in the aseptic loosening group had diabetes, compared to 27 of 352 patients (8%) in the control group (OR = 2.23, 95% CI 1.14–4.55, *P* = 0.02). After controlling for confounders using a multivariate logistic regression model, the association remained statistically significant (OR = 2.78, 95% CI 1.31–5.92, P = 0.01).

The odds of obesity were similar between the two groups (Table 2). Twenty-four (27%) were obese in the aseptic loosening group and 98 (28%) were obese in the control group (OR = 0.70, 95%CI 0.26–1.86, *P* = 0.32).

The prevalence of cardiovascular diseases was similar between cases and controls (Table 2). Of the patients in the aseptic loosening group, 23 (26%) had cardiovascular disease and 81 (23%) had cardiovascular disease in the control group (OR = 1.18, 95% CI 0.69–2.02, *P* = 0.31).

Respiratory diseases were also equally prevalent between the two groups (Table 2). Nine (10%) of aseptic loosening patients had respiratory diseases and 37 (11%) of control patients had respiratory diseases (OR = 0.97, 95% CI 0.45–2.09, *P* = 0.56).

Renal diseases were rare in our population in both aseptic loosening patients and controls (Table 2). Two patients (2%) had renal disease in the aseptic loosening group and eight patients (2%) in the control group, (OR = 1.0, 95%CI 0.21–4.80, *P* = 0.68).

Autoimmune diseases were also rare in our two groups (Table 2). Three patients (3%) had autoimmune conditions in the aseptic loosening group and 13 patients (4%) had autoimmune conditions in the control group, (OR = 0.92, 95%CI 0.26–3.30, *P* = 0.60).

**Table 2:** Patient related risk-factors for aseptic loosening

Disease category	Aseptic loosening (N = 88)	Control (N = 352)	OR	95% CI	*P*-value
Diabetes	14 (16%)	27 (8%)	2.28	1.14–4.55	0.02
Obesity	24 (27%)	98 (28%)	0.70	0.26–1.86	0.32
Cardiovascular	23 (26%)	81 (23%)	1.18	0.69–2.02	0.31
Respiratory	9 (10%)	37 (11%)	0.97	0.45–2.09	0.56
Renal	2 (2%)	8 (2%)	1.0	0.21–4.80	0.68
Autoimmune	3 (3%)	13 (4%)	0.92	0.26–3.30	0.60

Note: the variables included in the logistic regression model were age, sex, BMI and joint replaced

## Discussion

Our results demonstrate that patients undergoing revision arthroplasty for aseptic loosening have 2-fold greater odds of having a diagnosis of diabetes mellitus compared to controls. This adds to the body of evidence that there could be an association between aseptic loosening and diabetes mellitus.

Diabetes mellitus is a systemic disease which affects multiple organ systems, including bones. It can negatively affect bone mineral density, increase the risk of fractures and impairs fracture healing [16]. A large prospective cohort study identified the relative risk of hip fracture was 2.2 for type 2 diabetics and 6.4 for type 1 diabetics [18]. Similarly, in a meta-analysis of nearly 7 million patients, the relative risk of sustaining a hip fracture was 2.1 in diabetic patients [19]. Diabetics are also at a greater risk of non-union. A recent meta-analysis revealed an odds ratio of 2.1 for non-union in diabetic patients compared to non-diabetic patients [20]. The reasons for poor bone health in diabetic patients are multifactorial. Diabetics are predisposed to decreased bone mineral density which leads to a higher risk of fractures due to chronic inflammatory changes [21]. This chronic inflammation, in addition to glycation end products and formation of reactive oxygen species leads to the activation of the RANKL/OPG pathway, which increases osteoclast differentiation. Furthermore, it inhibits the differentiation of osteoblasts and increases osteoblast apoptosis [22]. This ultimately results in more bone resorption and decreased bone formation [23].

There are limited studies to our knowledge that investigate the links between diabetes and aseptic loosening. A large series of patients undergoing total knee arthroplasty demonstrated that those with diabetes had a significantly greater incidence of aseptic loosening (3.6% vs. 0.4%, *P* < 0.05), however, there were only 22 patients who had aseptic loosening in this case series [17]. Another large case series reported no significant difference in the risk of aseptic loosening in diabetics but found that hyperglycaemia one day preoperatively was associated with a greater risk of aseptic loosening (HR 4.95). However, this value was derived from only 11 patients who had aseptic loosening, again a relatively small sample [3]. Other studies have failed to demonstrate any significant difference between the risk of aseptic loosening in diabetics and non-diabetics [24–27]. Although the evidence linking diabetes to aseptic loosening is sparse and at times conflicting, the mechanisms of aseptic loosening certainly overlap with the inflammatory effects of diabetes on bone. Both aseptic loosening and diabetic related bone pathology ends in the final common pathway of increasing osteoclastic activity and decreasing osteoblastic activity [6, 23].

The link between the two disease processes is yet to be fully understood, and it is likely a very complex interaction. One potential mechanism could be a compounding effect of local inflammation due to wear debris from the prosthesis in addition to the systemic inflammatory effects of diabetes mellitus. Several cytokines have been implemented in both disease processes and may be the key to understanding the association between diabetes and aseptic loosening. These include TNF-α, IL-1α, IL-1β, IL-6, RANKL and PGE_2_ [6, 23, 28–34]. Perhaps the release of these cytokines into the systemic circulation in diabetics further potentiates their local effect in the bone-prosthesis interface, activating osteoclasts and inhibiting osteoblasts.

The limitations of our study must be acknowledged when interpreting our results. Firstly, this study is retrospective in nature and thus has all the limitations that are inherent to any retrospective study, including selection bias, recall bias and incomplete medical records. Secondly, our sample size is small, which could skew our findings in either direction and potentially underestimate or overestimate the associations identified. We note that the age of patients in our revision cohort is relatively young, which could be a potential confounder for aseptic loosening. Our patients did not have routine blood glucose levels measured and therefore this was not able to be defined. Several other factors were poorly documented in the clinical notes, which could be sources of bias in our results. These include the time from index operation, type of diabetes treatment, treatment compliance, smoking, cancer and vascular complications. Furthermore, we were unable to stratify patients based on type 1 or type 2 diabetes given the low numbers in our study. Future research could be aimed at exploring the association between diabetes and aseptic loosening further. This could be done either by expanding the sample size, such as conducting a registry-based study on a contemporary patient cohort. Another clinical study could investigate the effect of elevated blood glucose levels in patients with known and/or unknown diabetes mellitus, to determine whether blood glucose directly affects risk of revision. Basic science research can also be done to further define the potential mechanisms by which aseptic loosening could be worsened by diabetes mellitus.

## Conclusions

Our results demonstrate that there is a potential association between aseptic loosening and diabetes mellitus. This information can be used as a stimulus for future research. It can also be helpful in counselling diabetic patients undergoing primary joint replacement to educate them about their risk of aseptic loosening and other complications.

## Data Availability

The datasets used and/or analysed during the current study are available from the corresponding author on reasonable request.
